# Silk as a Natural Reinforcement: Processing and Properties of Silk/Epoxy Composite Laminates

**DOI:** 10.3390/ma11112135

**Published:** 2018-10-30

**Authors:** Youssef K. Hamidi, M. Akif Yalcinkaya, Gorkem E. Guloglu, Maya Pishvar, Mehrad Amirkhosravi, M. Cengiz Altan

**Affiliations:** 1 Mechanical Engineering Program, University of Houston−Clear Lake, Houston, TX 77058, USA; 2 School of Aerospace and Mechanical Engineering, University of Oklahoma, Norman, OK 73019, USA; akifyalcinkaya@ou.edu (M.A.Y.); gguloglu@ou.edu (G.E.G.); pishvar@ou.edu (M.P.); mehrad@ou.edu (M.A.); altan@ou.edu (M.C.A.)

**Keywords:** epoxy, natural fiber composites, silk fibers

## Abstract

With growing environmental awareness, natural fibers have recently received significant interest as reinforcement in polymer composites. Among natural fibers, silk can potentially be a natural alternative to glass fibers, as it possesses comparable specific mechanical properties. In order to investigate the processability and properties of silk reinforced composites, vacuum assisted resin transfer molding (VARTM) was used to manufacture composite laminates reinforced with woven silk preforms. Specific mechanical properties of silk/epoxy laminates were found to be anisotropic and comparable to those of glass/epoxy. Silk composites even exhibited a 23% improvement of specific flexural strength along the principal weave direction over the glass/epoxy laminate. Applying 300 kPa external pressure after resin infusion was found to improve the silk/epoxy interface, leading to a discernible increase in breaking energy and interlaminar shear strength. Moreover, the effect of fabric moisture on the laminate properties was investigated. Unlike glass mats, silk fabric was found to be prone to moisture absorption from the environment. Moisture presence in silk fabric prior to laminate fabrication yielded slower fill times and reduced mechanical properties. On average, 10% fabric moisture induced a 25% and 20% reduction in specific flexural strength and modulus, respectively.

## 1. Introduction

During the last several decades, fiber-reinforced polymer composites have experienced remarkable growth in various sectors, ranging from packaging and sporting goods to automotive and aerospace industries. This increased usage is essentially due to their lightweight, higher mechanical properties, and superior corrosion resistance compared to conventional materials [1,2].

Recently, growing environmental awareness has led to stricter policies regarding sustainability and encouraged industry to pursue ecofriendly products [3,4,5]. In this context, natural fibers have attracted increased attention over the past several years as alternatives to traditional reinforcements, namely glass, carbon, and aramid fibers. Currently, glass fibers are the most commonly used reinforcement in composites [3], since they offer a stable supply chain and relatively low-cost products with high mechanical performance. However, these inorganic fibers introduce several drawbacks, including non-biodegradability, high abrasion of processing equipment, and potential dermal and respiratory irritations [6]. In contrast, natural fibers offer a lower density, less abrasiveness, as well as promising biodegradability and sustainability [4,5,7]. For instance, plant-based natural fibers such as sisal, flax, jute, and hemp have been widely investigated in the literature as potential low-cost, ecofriendly alternatives to synthetic fibers [5,6,7]. Nonetheless, composites reinforced with plant fibers exhibit lower mechanical performance compared to those reinforced with glass fibers, which limits their use in structural applications [4,8,9]. Furthermore, plant fibers tend to exhibit thermal instability at elevated temperatures, lower impact strength, and mechanical degradation during processing. Despite these drawbacks, the commercial use of plant fiber composites in non-load-bearing applications has significantly increased, predominantly in the automotive industry [3,10].

In contrast to fibers extracted from plants, silk is an animal-based fiber that offers attractive features such as low density, flame resistance, and high elongation even at low temperatures [4,8,11]. More importantly, silk exhibits higher mechanical performance than plant fibers, and, in some cases, comparable specific mechanical properties to glass fibers [4,8,9]. Silk denotes a group of protein-based fibers, called fibroin, produced by several arthropods like silkworms, spiders, and scorpions [3,12]. Fibroin generally has an irregular, almost-triangular cross-section, with a width in the range of 8 to 13 µm [13]. Owing to its biocompatibility and bioresorbable properties as well as high strength and toughness, silk fibers are used in a variety of clinical applications, such as braided suture threads for surgical procedures and scaffolds for cartilage and bone repair [13,14]. Aside from biomedical applications, silk from the cocoons of the domesticated mulberry silkworm, *Bombyx mori*, is of particular interest in textile industry due to its availability [9,14,15]. Silk cocoons are generally degummed, spun into rovings and yarns, then woven into textile fabrics [14,15]. These woven silk fabrics may be used as a woven reinforcement in composites for structural applications [14,15,16]. In addition, some researchers have explored using abundant silk waste from textile industry to reinforce polymer composites [17,18,19].

Despite these promising features, silk fibers have received only limited interest as a reinforcement for polymer composite products, and practically no commercial use exists beyond biomedical applications [4,9,11,13,15]. One plausible explanation for this limited use is the higher cost of silk compared to plant fibers in a very cost-competitive environment, especially for nonstructural composite parts. While silk might be more expensive than conventional reinforcements, waste silk fabric can be processed and utilized in composite laminates in a cost-effective manner [17,18,19]. Another possible limiting factor of silk fibers seems to be the incompatibility between the hydrophilic natural fibers and the hydrophobic polymer matrix that requires some form of surface treatment to improve the interfacial bonding [3,6]. In addition, silk is known to be prone to environmental factors, such as moisture and UV radiation, that significantly alter the mechanical performance of the fibers [20,21]. Therefore, silk fibers and fabrics might require special storage and transportation conditions for best performance. Nevertheless, the high specific properties of silk fibers make it a suitable replacement for glass fibers in composite applications where lightweight and energy-absorbance are important, such as automotive, aerospace, and wind turbine structures [15,16].

The limited available literature on silk-reinforced composites mainly investigated either discontinuous silk fiber reinforced thermoplastics, or continuous silk fiber reinforced thermosetting composites. Due to their recyclability, discontinuous natural fibers, also referred to as short fibers, were traditionally used to reinforce injection-molded thermoplastics, particularly polypropylene [22]. More recently, short fiber silk composites were used to reinforce biodegradable thermoplastics, such as polylactic acid (PLA) [22], poly vinyl alcohol (PVA) [23], and polybutylene succinate [24]. For instance, Ho et al. [22] manufactured PLA composite reinforced with 5 wt.% short silk fibers by injection molding, and reported a 27% and 2% improvement over PLA in tensile and flexural moduli, respectively. Considerably higher improvements were reported in several mechanical properties for silk reinforced gelatin composites over neat gelatin [25], including a 260% increase in tensile strength, a 4-fold rise in tensile modulus, a 320% improvement in bending strength, a 450% increase in bending modulus, and a 260% improvement in impact strength. Although these improvements achieved using silk fibers are significant for certain ecofriendly applications, the obtained mechanical performance remains inferior to glass reinforced composites.

On the other hand, structural composite laminates, intended for energy-absorbing structures, are often fabricated using woven textile fabrics and stiffer thermosetting polymers [3,8,9,15,26,27]. Epoxy resins are frequently used owing to their lower cost, higher processability, higher mechanical properties, good adhesive performance, and chemical resistance [11,15]. For instance, Oshkovr and coworkers attempted to use woven silk/epoxy composite square tubes as energy-absorbers and evaluated their crashworthiness [8,26]. However, catastrophic failures were reported under compression tests in both studies. Although impressive single fiber properties might be reported in the literature [3,8,21], the actual improvement over unreinforced epoxy may be limited by defects in the silk fabric, such as fiber misalignment and waviness inadvertently introduced during weaving. Furthermore, Yang et al. [15] investigated the tensile, flexural, interlaminar shear, impact, dynamic, and thermal properties of the silk/epoxy composites at 30%, 40%, 50%, 60%, and 70% fiber contents. A linear increase of most properties was observed with increasing fiber content between 30% and 70%. Optimal tensile properties were observed at 70% fiber content, with 145%, 130%, and 70% improvement over neat epoxy in tensile stiffness, ultimate stress, and ultimate strain, respectively. In addition, impact strength was observed to increase significantly only for fiber contents above 60%. The same research group studied silk/epoxy laminates for two natural silk varieties: *Bombyx mori* and *Antheraea pernyi* silk [27]. The authors reported that at 60% fiber content, both silk types showed a 2-fold increase in both specific tensile modulus and strength compared to the unreinforced epoxy resin. For the 60% *Antheraea pernyi* silk/epoxy laminates, the breaking energy was found to be 11.7 MJ/m^3^, an order of magnitude higher than the 1.1 MJ/m^3^ measured for neat epoxy. Moreover, a 3-fold increase in specific flexural strength was also reported, reaching 316 MPa/g·cm^−3^.

More recently, Shah et al. [3,7,10] attempted to make a case for silk as a reinforcing agent in composite laminates by comparing their mechanical performance to flax- and glass-reinforced composites. The authors fabricated silk/epoxy laminates with nonwoven silk preform at 36% fiber content and woven silk fabric at 45% fiber content. The authors reported tensile and flexural specific strengths of ~90 MPa/g·cm^−3^ and ~170 MPa/g·cm^−3^, respectively [3]. These values were comparable, although not necessarily superior, to those of glass/epoxy laminates. Other researchers incorporated silk into glass reinforced composites in the pursuit of hybrid composites with improved impact properties [16,17,28,29,30]. For example, Zhao et al. [16] investigated silk fabric/glass mat/polyester hybrid laminates at 14.5% and 2.4% fiber content of glass and silk, respectively. However, the authors reported practically no effect of the limited silk fabric presence on impact and flexural properties.

Surprisingly, two important aspects were not addressed in the available literature on silk reinforced composites. First, silk fabric is mostly used as received and surface treatment is seldom attempted to improve the silk/epoxy interface [31,32,33]. Generally, fiber sizing can be used to tune the bulk properties of composite laminates [34,35]. Surface treatment of natural fibers has been shown to significantly improve the properties of composite laminates [36,37,38,39]. Second, no attempt was made to investigate the effect of manufacturing processes and relevant process parameters on the produced silk composites. In fact, most of the reported investigations employed a rather simple, hand lay-up method to manufacture silk/epoxy laminates [16,17,30,40]. While simple fabrication methods such as hand lay-up can be attractive for their relative ease and low cost, they are operator-dependent, prone to process-induced defects, and often result in low-quality composite parts with higher void occurrence [40]. Presence of these defects, in turn, is known to significantly degrade the mechanical performance of composites [41]. A few other studies [9,15,27] used hand lay-up followed by hot pressing in order to increase the fiber content of the laminates, and thus improve the composite performance.

As described in the previous paragraph, remarkable improvements over neat epoxy were only achieved for silk/epoxy composites with fiber contents of 60% and higher [15,27]. Studies conducted at low or moderate fiber contents did not yield considerable improvement in mechanical performances. Consequently, investigating more appropriate manufacturing processes for silk/epoxy composite applications, such as variants of liquid composite molding (LCM), might be of interest. Only a couple of articles used vacuum-assisted resin transfer molding (VARTM) to manufacture silk/epoxy laminates [3,40]. In fact, Shah et al. [3] were able to achieve comparable mechanical performance to glass/epoxy laminates at a fiber content of only 45%. A lower occurrence of process-induced defects within the silk composites is believed to play a significant role in reaching this performance. More recently, our research group investigated fabrication challenges for silk/epoxy laminates [40], which showed that compared to hand lay-up, VARTM was more appropriate for silk/epoxy laminate fabrication as it allows uniform impregnation of the silk preform by the liquid resin, yielding higher part quality and reduced void formation.

In order to investigate the suitability of silk as an alternative reinforcement to glass fibers in polymer composites, the processability of silk reinforced composites was verified by fabricating silk/epoxy laminates using VARTM. In addition, the effects of manufacturing process and microstructural parameters such as post-fill external pressure and silk fabric anisotropy on the process-induced microstructure of silk epoxy laminates were studied. The mechanical performance of the fabricated laminates was also compared with those of neat epoxy and glass/epoxy laminates. Finally, the effect of storage conditions of silk fibers and moisture absorbed by silk on the manufacturing and performance of silk/epoxy composites was investigated.

## 2. Materials and Methods

### 2.1. Materials

INF 114 epoxy (PRO-SET) was used as the resin and polyamine INF 211 (PRO-SET) was chosen as the hardener. Before laminate fabrication, the resin and curing agent were mixed for 5 min at 350 rpm at a ratio of 100:27.4 by weight and degassed for 10 min under vacuum.

A woven silk fabric (Satin Ahimsa, Aurora Silk, Inc., Portland, OR, USA) was used in this study. The silk was produced from degummed cultivated *Bombyx mori* mulberry silk and had an areal density of 81.4 g/m^2^. For each laminate, twelve layers of 152 mm-wide × 203 mm-long (6″ × 8″) silk fabric were prepared, stacked, and placed on the mold before infusion. Preparation of silk layers involved cutting the fabric to the desired size, ironing to remove wrinkles, and then drying in a vacuum oven at 50 °C. Since silk fabric exhibited an unbalanced weave pattern, laminates were fabricated with layers cut along both planar directions to investigate the effect of fabric anisotropy on the fill time and mechanical performance. Hence, two separate sets of laminates were investigated for each case: one set with fabric layers cut such that its length is parallel to the roll direction (longitudinal), and another set cut such that its length is perpendicular to the roll direction (transverse).

### 2.2. Manufacturing Procedure

**Figure 1:**
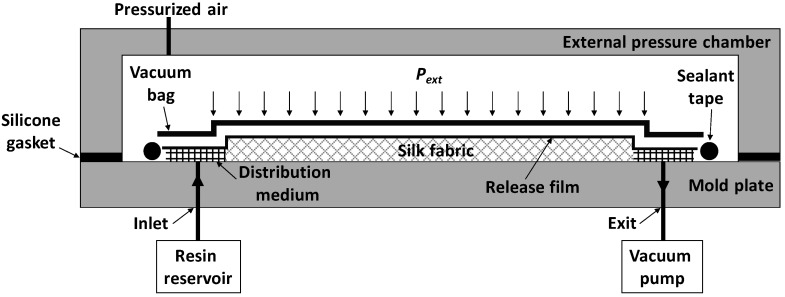
Experimental vacuum-assisted resin transfer molding (VARTM) setup with external pressure chamber to fabricate woven silk/epoxy composite laminates.

An improved variant of vacuum-assisted resin transfer molding (VARTM) was used to fabricate silk/epoxy laminates. Figure 1 illustrates the experimental molding setup which can facilitate external air pressure on a typical VARTM mold to increase the limited compaction pressure in conventional VARTM (i.e., higher than 1 atm) [42]. Depending on the applied pressure, the fiber volume fraction in fabricated laminates can be significantly increased, which yields high-quality laminates with improved mechanical properties.

As seen in Figure 1, 12 layers of woven silk fabric (i.e., preform) were sealed with a vacuum bag and the epoxy/hardener mixture was infused into the mold from the inlet resin reservoir towards the vacuum source (i.e., exit). In order to reduce the effect of ambient temperature fluctuations, the mold temperature was kept constant at 30 °C. At this temperature, the viscosity of the resin was in the range of 180 to 200 mPa s. After the preform was completely wetted, infusion was continued for an additional 5 min (i.e., resin flushing) to mobilize and remove the process-induced voids with resin outflow from the exit gate [42]. Once the resin flushing was completed, the inlet port was closed to remove the excess resin from the exit of the mold (i.e., resin bleeding). In certain fabrication scenarios an external chamber pressure of 300 kPa (~44 psi) was applied during the post-filling stage to further compact the preform and improve the mechanical properties. All fabricated laminates were cured at 60°C for 8 hours after resin gelation occurred in 5 h at 30 °C.

Table 1 presents the various fabrication scenarios used in this study and lists the laminate designations, reinforcement types, and fabric/lay-up details as well as the thicknesses of the fabricated laminates. For comparison purposes, neat epoxy samples (E) were manufactured by gravity casting and cured following the same cure schedule. In addition, glass/epoxy laminates (G), fabricated using the same resin in a recent study by our research group [43], were considered. Five different fabrication scenarios for silk composites (S) were performed. These scenarios were designed to assess the effects of fabric anisotropy, external pressure, and fabric moisture on the properties of the fabricated laminates. Furthermore, fabric anisotropy effects on wetting characteristics such as fill time, critical in defining the manufacturing cycle, were also investigated.

**Figure 2:**
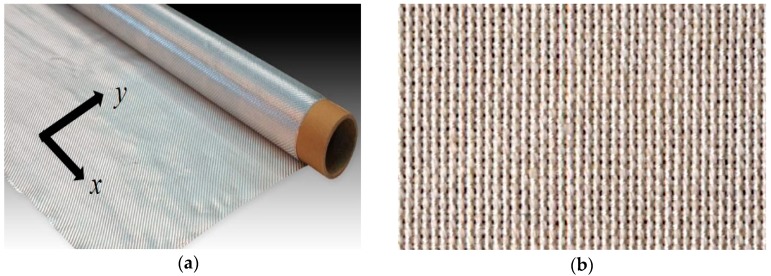
Representative images of the fabric, showing (**a**) longitudinal (abbreviated L for filling and *y* for testing) and transverse (abbreviated T for filling and *x* for testing) directions with respect to fabric roll and (**b**) a macroscopic image of the weave pattern.

Figure 2a shows a representative image of the fabric and the disposition of longitudinal (*y*) and transverse (*x*) directions with respect to the fabric roll. Additionally, Figure 2b shows that the silk fabric was tightly woven with low porosity between the fibers with several silk threads in orthogonal directions. Abbreviations “L” and “T” were used to indicate whether the infusion was performed along the longitudinal or transverse directions of the fabric roll, respectively. In contrast, symbols *x* and *y*, depicted in Figure 2, were exclusively used in the designation to indicate testing directions of the composite samples. In addition, “P” indicates that external pressure was applied during the post-filling stage. “M” designates that the silk fabric was exposed to moisture prior to molding. For each fabrication scenario, two identical, 152 mm × 203 mm laminates were fabricated to ensure the repeatability of fabrication procedure.

### 2.3. Sample Preparation

Each molded laminate was sectioned using a diamond saw into several rectangular specimens. According to ASTM standards (D792, E1131, D790, D7028, and D2344), five samples for density measurement, five for thermogravimetric analysis, twelve for flexural testing (six samples along the flow direction (*y*), and six along (*x*) perpendicular to the flow), ten for SEM imaging (five along and five perpendicular to the flow direction) were cut in particular dimensions. When the sample thickness allowed, ten rectangular samples were cut for short beam tests (five along and five perpendicular to the flow direction).

### 2.4. Density Measurement and Volume Fraction Determinations

The density of five samples from each laminate was measured according to ASTM D792. Similarly, the density of the neat epoxy (i.e., the matrix) was measured as *ρ_Epoxy_* = 1.140 ± 0.001 g/cm^3^. The density of silk fibers, on the other hand, was measured using a gas pycnometer (AccuPyc II 1340) as *ρ_Silk_* = 1.361 ± 0.002 g/cm^3^. Using both densities of silk and epoxy, as well as the measured density of each fabricated composite laminate, *ρ_Laminate_*, both fiber volume fraction, *V_f_*, and resin volume fraction, *V_r_* can be calculated as

(1) $$V_{f}=\frac{\rho_{Laminate}\;M_{Silk}}{\rho_{Silk}\;M_{Laminate}}$$

(2) $$V_{r}=\frac{\rho_{Laminate}}{\rho_{Epoxy}}\times \frac{M_{Laminate}-M_{Silk}}{M_{Laminate}},$$

where *M_Laminate_* is the measured mass of the laminate and *M_Silk_* is the measured mass of the twelve silk fabric layers. Using both fiber and resin volume fractions, void volume fraction, *V_ν_*, can be calculated for each laminate as

(3) $$V_{v}=1-(V_{f}+V_{r})$$

### 2.5. Thermogravimetric Analysis

Thermogravimetric analysis (TGA) was used to verify the thermal stability and identify the maximum temperature at which the silk/epoxy composite laminates can be used. TGA thermograms were obtained by using a Thermogravimetry-Differential Scanning Calorimetry (TG-DSC) instrument (TA Instruments Q50, New Castle, DE, USA) at 10 °C/min heating rate in a nitrogen atmosphere.

### 2.6. Mechanical Testing

Flexural tests were performed according to the ASTM D790 standard to measure the flexural properties of each laminate. As mentioned earlier, specimens from each laminate were used to characterize the flexural properties along the longitudinal (*y*) and transverse (*x*) directions. Short beam tests were also executed on rectangular samples along the *y*- and *x*-directions cut from each silk/epoxy laminates. Interlaminar shear strength (ILSS) values were obtained in accordance with ASTM D2344. The mechanical testing was carried out at a rate of 2 mm/min.

### 2.7. Dynamic Mechanical Analysis

A dynamic mechanical analyzer (DMA) (TA Instruments DMA-Q800, New Castle, DE, USA) was used to measure the glass transition temperature, *T_g_*, of selected configurations of the composites, namely neat epoxy (E), glass/epoxy (G), silk/epoxy impregnated along the transverse direction (S/T), and moist silk/epoxy impregnated along the transverse direction (S/T/M). For these particular measurements, 51 mm × 12.7 mm (2″ × 0.5″) specimens were prepared. The storage modulus, loss modulus, tanδ, and glass transition temperatures were determined for each sample.

### 2.8. Scanning Electron Microscopy (SEM) Imaging

SEM imaging was performed for the analysis of the microstructure of composite laminates. Specimen cut from the laminates were mounted in an acrylic resin to expose the through-the-thickness cross-section. Once polished, the specimens were sputter coated with 5 nm of gold/palladium to avoid charge build-up during SEM imaging. SEM images at different magnifications were obtained using a Zeiss Neon 40 EsB microscope (Carl Zeiss AG, Oberkochen, Germany). Additionally, fracture surfaces of the flexural test samples, as well as the silk preforms, were examined by SEM.

## 3. Results and Discussion

### 3.1. Morphology and Microstructure of Silk Fabric

Specialty material suppliers for composite reinforcement usually provide fabrics with balanced weave patterns, thus practically ensuring that the longitudinal and transverse properties would be similar. Having a fabric with a balanced planar weave fabric also ensures similar impregnation dynamics along both principal fabric directions. On the other hand, commercial fabrics for textile applications generally exhibit unbalanced weave patterns. For instance, the SEM micrograph of the silk fabric used by Yang et al. [15] showed a clearly unbalanced weave. Therefore, the morphology and microstructure of the silk fabrics that are often used for textile applications need to be investigated in detail to reveal possible anisotropy in their weave patterns.

**Figure 3:**
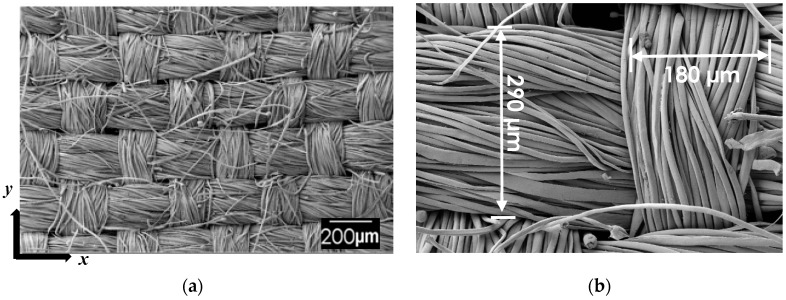
Sample SEM images of the microstructure of silk fabric, showing (**a**) an SEM image of multiple yarns (50×) and (**b**) higher magnification SEM image of two orthogonal yarns (200×).

SEM images presented in Figure 3 depict the microstructure of the silk fabric used in this study. Figure 3a shows the disposition of silk yarns along the longitudinal (*y*) and transverse (*x*) directions with respect to the fabric roll. The weave pattern was observed to be anisotropic, as clearly shown in Figure 3a,b. In fact, yarns along the transverse (*x*) direction have an average width of ~290 µm, which is approximately 60% higher than its counterpart along the longitudinal (*y*) direction (~180 µm). This unbalance in the weave pattern will likely induce disparate filling patterns and anisotropy in mechanical performance along these orthogonal directions.

### 3.2. Morphology and Microstructure of Silk/Epoxy Composite Laminates

**Figure 4:**
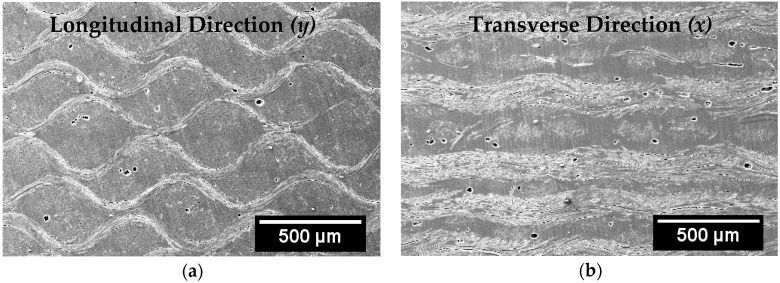
Representative SEM images of the through-the-thickness microstructure of fabricated silk/epoxy composite laminates: (**a**) along the longitudinal (*y*) direction and (**b**) along the transverse (*x*) direction. Both images are at 30×.

Figure 4 depicts representative SEM images of the through-the-thickness microstructure of the fabricated silk/epoxy laminates. Figure 4a is a representative image along the longitudinal direction (*y*), obtained at 30×. Figure 4b, on the other hand, shows a representative image along the transverse direction (*x*) obtained at the same magnification. On these micrographs, fiber tows can be seen in both parallel and perpendicular directions to the cross-section. While a good wetting of the silk fabric was observed for all considered cases, voids are occasionally observed within the resin. Void occurrence was observed to be lower for laminates fabricated with external pressure. Interestingly, tows along the *x* and *y* directions showed contrasting undulation through-the-thickness of the composite. Figure 4a shows a pronounced sinusoidal waviness of silk tows continuously along the longitudinal direction, while tows along the transverse direction were less wavy (Figure 4b). Fiber waviness is known to have a substantial influence on the mechanical performance of the composites [41], thus mechanical properties were expected to be significantly lower along the longitudinal direction compared to the transverse direction.

### 3.3. Thermal Stability of Silk/Epoxy Composite Laminates

**Figure 5:**
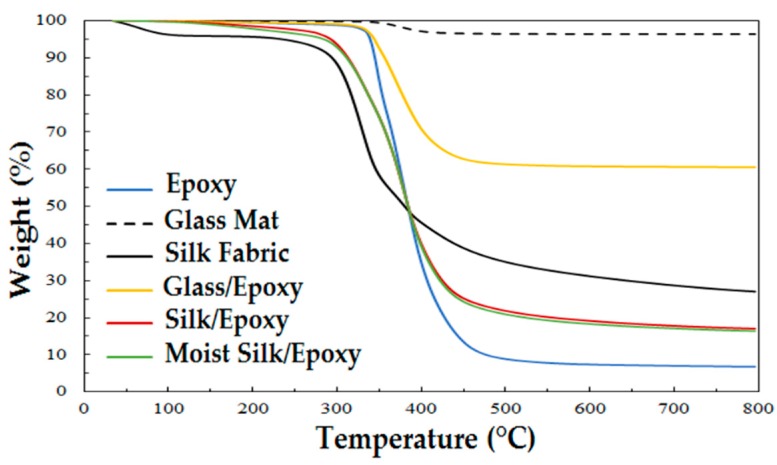
TGA thermographs of neat epoxy, glass mats, silk fabric, as well as glass/epoxy and silk/epoxy laminates, under a nitrogen atmosphere at a heating rate of 10 °C/min.

Figure 5 shows TGA thermograms of neat epoxy, silk and glass fibers, in addition to the different composite laminates. The weight of neat epoxy was practically constant below 350 °C. Thermal decomposition of epoxy occurred mostly between 350 °C and 500 °C, after which no significant change in weight was observed. For silk fibers, no discernable change was observed at the processing temperature of 30 °C, indicating that the properties of silk fibers would not be altered during manufacturing. Furthermore, an initial weight loss of ~5%–10% was observed below 100 °C, which is believed to correspond to desorption of moisture absorbed by the silk fabric from the environment. Thermal decomposition of the silk started at ~300 °C and continued at a decreasing rate until ~800 °C. In contrast, glass fibers showed a minor thermal decomposition between ~350 and ~450 °C, after which glass fiber weight did not change up to ~800 °C. This slight weight loss is possibly due to the removal of the organic sizing on the glass fibers. The divergence in thermal degradation behaviors of the three composite constituents would originate from the dissimilarities in their chemical compositions as well as their surface treatments.

Figure 5 also presents TGA spectra of both glass/epoxy and silk/epoxy laminates. For both composite types, the thermal degradation pattern showed three main stages. The first stage took place before thermal degradation occurred, where the weight was fairly stable. The second stage started with the decomposition of organic constituents of the composite. For G laminates, this stage started at ~350 °C, initiated by the degradation of the neat epoxy. Similarly, S laminates decomposition started at ~300 °C concurrently with the degradation of silk fibers. For both G and S composites, the thermal decomposition continued until ~450 to 500 °C due to the combined decomposition of the epoxy matrix and either the silk fibers or the sizing of glass fibers. In the final stage up to ~800 °C, the weight loss was observed to be minimal. These results showed that no thermal degradation was observed for silk/epoxy laminates up to ~300 °C, which was almost comparable to that of glass/epoxy laminates.

### 3.4. Fabric Anisotropy Effects on Fiber Wetting

The anisotropy of the silk fabric significantly influenced the impregnation characteristics during fabrication by VARTM. As presented in Table 2, impregnation along the transverse (*x*) direction was found to be much faster with an average filling time of 20 min. In contrast, when the impregnation was along the longitudinal (*y*) direction of the fabric roll, an average fill time of 68 min was observed. This ~140% increase in fill time was attributed to the anisotropic permeability of the silk fabric. As shown in Figure 3, fiber tows were ~60% thicker along the transverse direction than the longitudinal direction. This difference in yarn distribution would induce contrasting preform fabric permeabilities along the transverse and longitudinal directions, yielding this substantial increase in fill time.

The fabric anisotropy not only affected the mold filling time but also the final void content in the fabricated laminates as the impregnation velocity significantly affect the void formation [44]. As Table 2 documents, slow impregnation while fabricating the S/L and S/L/P laminates yielded lower void contents than those impregnated more rapidly (i.e., S/T and S/T/P, respectively). For instance, silk/epoxy laminates impregnated along the longitudinal direction (S/L) filled in 68 min and yielded a void content of 0.7%. In contrast, the laminates impregnated under identical conditions along the transverse direction (S/T) filled in 20 min, with a void content of 1.1%. A similar trend was observed for laminates manufactured with external pressure, as void contents of 0.5% and 1.2% observed for S/L/P and S/T/P, respectively. In LCM processes, voids are known to form mainly when air is trapped due to the unbalance between the velocities of the viscous macroflow occurring between fiber tows and the capillary flow inside fiber tows [44]. According to the modified capillary number analysis, any change in the equilibrium between these competing flows would affect the air entrapment void formation mechanism, thus affecting void presence in the final composite part [1]. The highest void content was observed for the fastest filling of 3 min corresponding to the glass/epoxy laminate case. Nonetheless, the comparison does not hold as the sized glass fibers possess an entirely different epoxy affinity and the glass fabric is in the form of a random mat.

Furthermore, slower filling slightly increased the amount of resin intake during mold filling, resulting in a slight drop in fiber content (from 44% to 43% for S/T and S/L, respectively). Applying a post fill external pressure helped improve the fiber content in silk laminates from ~43% to ~47%. It is worth noting that the increase in fiber volume fraction induced a slight increase in the density of the composite laminate. Applying external pressure after the impregnation is completed, i.e., after formation of voids, can have opposing effect on the final void content of the part. On one hand, it helps in suppressing voids due to increased pressure and, on the other hand, it may cause void entrapment and prevent removal of mobile voids during flushing or bleeding of the resin. As a result, when impregnation was performed along the longitudinal direction, applying 300 kPa pressure reduced void content from 0.66% to 0.53%. However, in the case that impregnation was along the transverse direction, void content increased from 1.12% to 1.24%.

### 3.5. Fabric Anisotropy Effects on Mechanical Properties of Silk/Epoxy Laminates

**Figure 6:**
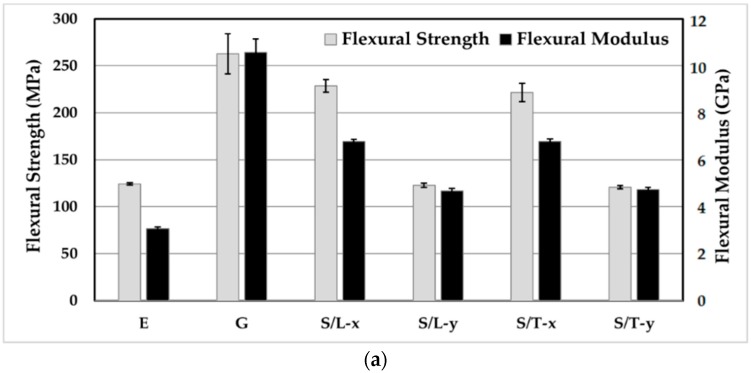

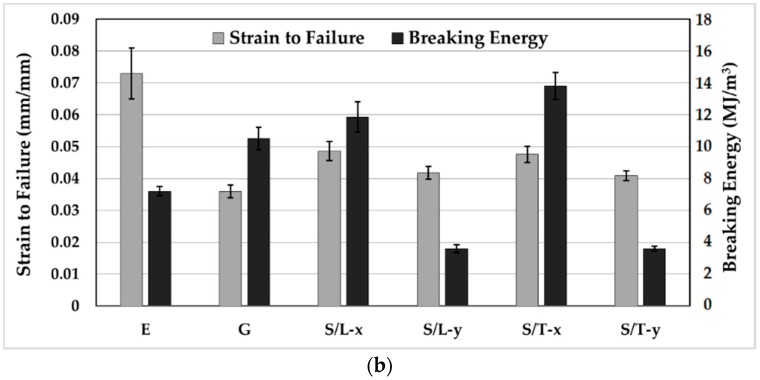
Flexural properties of silk/epoxy composite laminates in comparison with neat epoxy and glass/epoxy laminates [43]: (**a**) Flexural strength and modulus and (**b**) strain to failure and breaking energy.

Figure 6a shows both flexural strength and modulus of neat epoxy, random mat glass/epoxy laminates, and silk/epoxy laminates infused along both the longitudinal and transverse directions, each tested along orthogonal directions *x* and *y*. Moreover, Figure 6b depicts strain to failure and the breaking energy calculated as the area below flexural stress-strain curves of the tested specimen.

A strong anisotropy was observed in the flexural performance of silk/epoxy laminates. In fact, flexural strength, modulus, and breaking energy were observed to show disparate properties when tested along the longitudinal and transverse directions. As depicted in Figure 6a, flexural strength dropped by 46% from 229 MPa along the *x* direction to 123 MPa along the *y* direction for the silk/epoxy laminate infused along the longitudinal direction. A similar drop of 46% from 222 to 121 MPa was observed when infusing along the transverse direction. Flexural modulus showed an analogous tendency with 31 and 30% reductions between the *x* and *y* directions when infused along the longitudinal and transverse directions, respectively. Furthermore, Figure 6b depicts a similar trend for the breaking energy. For the silk epoxy laminate infused along the transverse direction (S/T), breaking energies of 13.8 and 3.6 MJ/m^3^ were calculated respectively along the *x* and *y* directions, representing an almost 74% difference. Similarly, a 70% difference (11.8 to 3.6 MJ/m^3^) was observed in the breaking energy for S/L along the longitudinal direction. Strain to failure is the flexural property least affected by the fabric microstructure with only 14% difference along the *x* and *y* directions in both S/T and S/L.

As shown in Figure 3, yarns along the transverse (*x*) direction are ~60% thicker than yarns along the longitudinal (*y*) direction, which implies a larger number of silk fibers would be under loading along the *x* direction compared to those along the *y* direction. Furthermore and as shown in Figure 4a, higher waviness of the silk tows throughout the laminate was observed in the longitudinal (*y*) direction. Localized wrinkles are known to dramatically reduce the composite mechanical properties depending on the wrinkle severity, defined as the ratio of the amplitude to the wavelength of the undulation [41]. The wrinkles of the silk fabric along the *y* direction shown in Figure 4a exhibited a wrinkle severity in the range of 0.25 to 0.30. Single wrinkles with a severity in that range are reported to induce as much as 80% reduction in mechanical properties [41]. Continuous undulations are known to cause even further deterioration of composite performance [41]. In a sense, the undulated silk fabric along the longitudinal direction may be acting more as a defect rather than a reinforcement.

The results given in Figure 6 indicate that the mechanical performance of silk/epoxy laminates did not seem to depend on the infusion direction. In fact, regardless of the infusion direction, the laminates tend to exhibit statistically similar mechanical properties. The slight differences in void and fiber contents did not seem to affect the resulting flexural and breaking energy performance. For instance, the S/T laminate exhibited a ~44% fiber content, similar to the ~43% fiber content of S/L. A small difference in the void contents was observed in these laminates, with 1.1 and 0.7% void contents for S/T and S/L, respectively. Hence, the efficiency of manufacturing silk composites can be eventually improved in an industrial setting by an appropriate selection of infusion direction that reduces filling times and manufacturing cycles.

### 3.6. Silk/Epoxy Laminate Performance Compared to Neat Epoxy and Glass/Epoxy Laminates

In addition to silk/epoxy laminates, Figure 6 depicts the flexural performance of glass/epoxy laminates from a past paper [43] and neat epoxy. Silk was found to improve the flexural strength and modulus, as well as the breaking energy of epoxy along *x* direction. For instance, more than 78% and 121% improvements were obtained in flexural strength and modulus, respectively, compared to pure epoxy along *x* direction. In contrast, change in flexural strength was negligible along the *y* direction. Conversely, flexural modulus showed more than 53% increase over the neat epoxy along the *y* direction.

An expected drop in strain to failure was observed for silk/epoxy laminates compared to neat epoxy, as the addition of fibers limits the ability of the epoxy resin to elongate. However, the measured values of 0.041 to 0.049 were definitely higher than the strain to failure of 0.036 exhibited by glass/epoxy laminates. This result was also predictable as silk fibers enjoy a remarkably high failure strain of ~20% compared to the ~2.5% of glass fibers [3,8,9]. Lai and Goh reported an even higher strain to failure of ~34% [21]. These large failure strains would allow the silk reinforced laminates to experience higher failure strains compared to glass/epoxy composites, yielding improved breaking energy.

Aside from strain to failure, the glass/epoxy laminates performed slightly better than silk/epoxy at comparable fiber contents (~45%) in terms of flexural properties. Along the *x* direction, silk composites enjoyed flexural strengths merely 12 to 15% lower than glass laminates, as shown in Figure 6a. Similarly, silk flexural moduli were ~36% lower than glass/epoxy laminates. In contrast, and owing to the superior elongation of silk, the breaking energy of silk/epoxy were 35% higher than that of glass/epoxy laminates.

**Figure 7:**
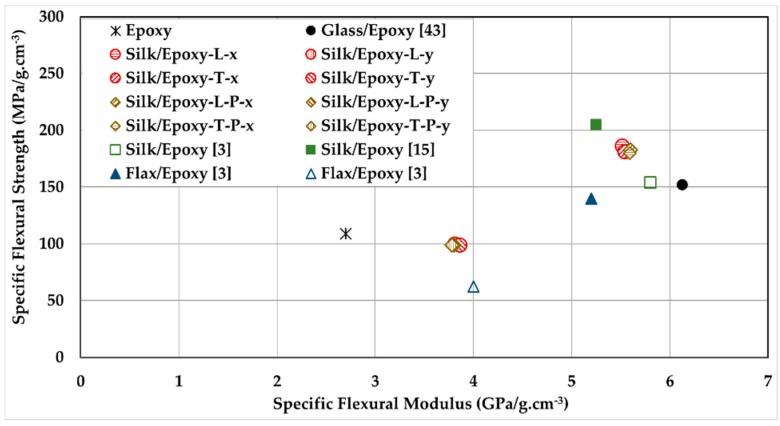
Specific flexural properties of silk/epoxy laminates compared to glass/epoxy laminates and neat epoxy samples, as well as reported properties for silk/epoxy and flax/epoxy from the literature.

In high-end applications where both lightweight and high performance are pursued, specific properties are often used to assess the composite parts [3,15]. Figure 7 illustrates the specific flexural strength vs. specific flexural modulus for the silk/epoxy and glass/epoxy composites investigated in this study. For comparison purposes, results reported in the literature for silk/epoxy laminates [3,15] and flax/epoxy laminates [3] with comparable fiber contents are also included.

First, as shown in Figure 7, the measured specific flexural strength and modulus compared well with the values reported in the literature for silk/epoxy laminates [3,15], and performed better than flax/epoxy laminates [3]. It is worth noting that both references reported only the mechanical properties of silk/epoxy laminates along strongest direction, although the unbalanced weave patterns were observed in the silk fabric used in this study.

The lowest value of specific flexural strength of silk/epoxy in this study was measured at ~100 MPa/g·cm^−3^ along the *y* direction, which is slightly lower than unreinforced epoxy. In contrast, the specific flexural strength along the *x* direction exhibited more than 66% improvement over epoxy. Moreover, silk reinforcement induced ~41 and ~104% increases in specific flexural modulus along the *x* and *y* directions, respectively.

Compared to glass/epoxy, silk/epoxy laminates showed a 19 to 23% higher specific flexural strength along the *x* direction, and a concurrent ~10% lower specific modulus. This improvement in specific properties is significant, given the large difference between the properties of individual silk and glass fibers [4,8,9,15,16,21]. Along the *y* direction, the specific flexural strength and modulus were ~35 to 37% lower than glass/epoxy laminates. This was expected as the silk was not observed to effectively reinforce the epoxy along the *y* direction. Applying a post-fill external pressure did not seem to affect the specific flexural properties of the silk/epoxy laminates. The slight improvement in absolute flexural strength and modulus was offset by the concurrent increase in laminate density. These results showed that silk/epoxy may provide comparable, if not better, specific properties to glass/epoxy at least along the transverse direction. Once balanced weave patterns are produced without yarn undulations, silk fabrics can deliver the same reinforcement along both principal directions.

### 3.7. Silk/Epoxy Interface

**Figure 8:**
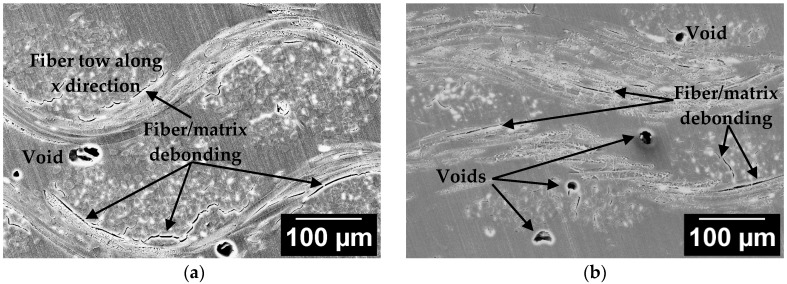
Sample SEM images at 200× of the microstructure of silk/epoxy laminate cross-sections (**a**) along the *y* direction and (**b**) along the *x* direction.

A possible explanation of the lower absolute flexural properties of silk/epoxy laminates compared to glass/epoxy laminates may be an inferior bonding between silk fibers and the epoxy matrix. In fact, unlike glass fibers that have a commercial sizing suitable for epoxy matrix bonding, the silk fabric was used as received and did not undergo any surface treatment. Thus, a weak fiber/matrix adhesion is expected due to the incompatibility between the hydrophilicity of untreated silk fibers and the hydrophobicity of the epoxy [3,6]. The silk/epoxy interface was investigated by the careful review of the SEM images of silk/epoxy laminate cross-sections, as depicted in Figure 8.

Inspection of the silk/epoxy interface under SEM showed extensive fiber/matrix debonding in all fabricated laminates, indicating an overall weak fiber/matrix adhesion. In addition, fiber/matrix debonding was observed to have a higher occurrence along the *y* direction, as depicted in Figure 8a,b. A weak fiber/matrix adhesion is expected due to the incompatibility between the hydrophilicity of untreated silk fibers and the hydrophobicity of the epoxy [3,6]. In addition, the pronounced undulations of silk yarns along the longitudinal (*y*) direction might increase the fiber/matrix debonding as the curvatures create a more tortuous path for the impregnating epoxy resin. Furthermore, fiber/matrix debonding was visually observed to be much less frequent in laminates manufactured with post-fill external pressure, as the application of external pressure might have increased the resin pressure, thus causing a reduction on the porosity as well as slightly improving the fiber/matrix interface [42,45].

**Figure 9:**
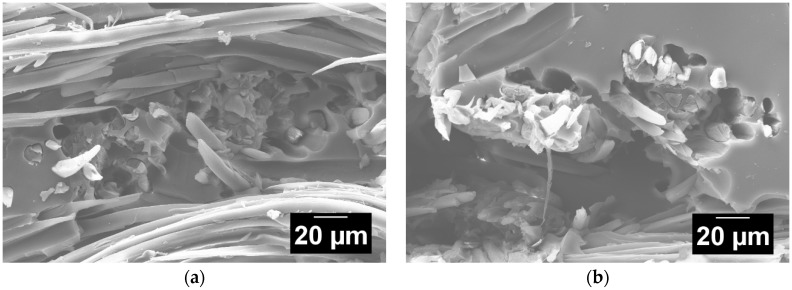
Sample SEM images at 400× of the fractured surfaces of silk/epoxy laminates fabricated (**a**) without external pressure and (**b**) with external pressure.

Another method to investigate the fiber/matrix adhesion is to analyze fracture surfaces of the silk/epoxy laminates. As illustrated in Figure 9a,b, fiber pullouts were observed in SEM images of fracture surfaces of tested silk/epoxy specimens, even in the laminates fabricated with external pressure. These findings corroborate the weak fiber/matrix adhesion and highlight the potential benefit of an appropriate surface treatment for silk fibers.

### 3.8. Effect of Post-Fill External Pressure on Silk/Epoxy Performance

The results presented earlier indicate that the external pressure did not affect the flexural properties of silk/epoxy laminates. Statistically indistinguishable values of strength, modulus, and strain to failure were measured with and without applying pressure for all considered cases. Specific flexural strength and modulus also showed no sensitivity to external pressure as the data points are observed to overlap in Figure 7. This insensitivity could be due to minor increases in fiber volume fraction at the external pressure level applied in this study, which could only yield a modest increase of ~4 to 5% in fiber content.

**Figure 10:**
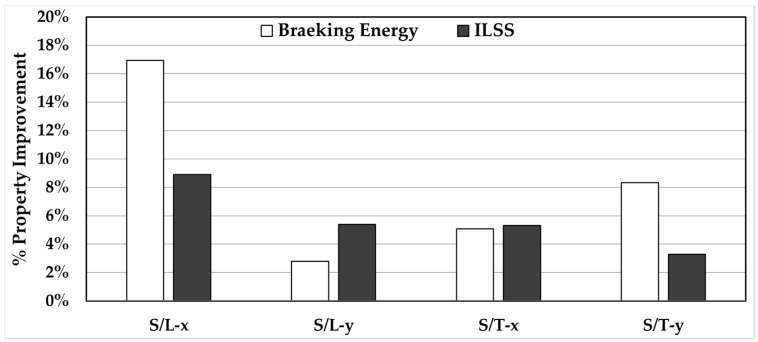
Effect of applying a 300 kPa post-fill external pressure to silk/epoxy laminates on the breaking energy and interlaminar shear strength (ILSS).

Breaking energy and interlaminar shear strength (ILSS), on the other hand, showed discernible improvement with the application of post-fill external pressure. Figure 10 presents the percent property improvement of both the breaking energy and ILSS due to the application of external pressure. For example, the energy required to break silk/epoxy laminates along the *x* direction increased by ~17% from ~12 MJ/m^3^ to ~14 MJ/m^3^ after applying the external pressure for the slow-filled silk/epoxy laminates infused along the longitudinal direction. Lower improvements (3%–8%) in breaking energy were observed for the remaining cases. In addition, Figure 10 shows a consistent improvement in ILSS (3%–9%) after the application of external pressure. Again, the highest improvement in ILSS (i.e., 9% to 44 MPa) was observed in the slow-filled silk/epoxy laminates along the *x* direction. These improvements in breaking energy and ILSS can be attributed to a lower occurrence of silk/epoxy debonding at higher pressures observed during SEM analysis.

### 3.9. Dynamic Mechanical Analysis of Silk/Epoxy

Dynamic mechanical analysis (DMA) is usually used to investigate the mechanical properties and viscoelastic behavior of polymer-based materials as a function of temperature, frequency, and time [15]. In this study, DMA experiments were performed for two purposes: (a) to characterize the thermomechanical properties of the silk/epoxy composite laminates and (b) to compare glass transition temperature of silk/epoxy and glass/epoxy composites.

**Figure 11:**
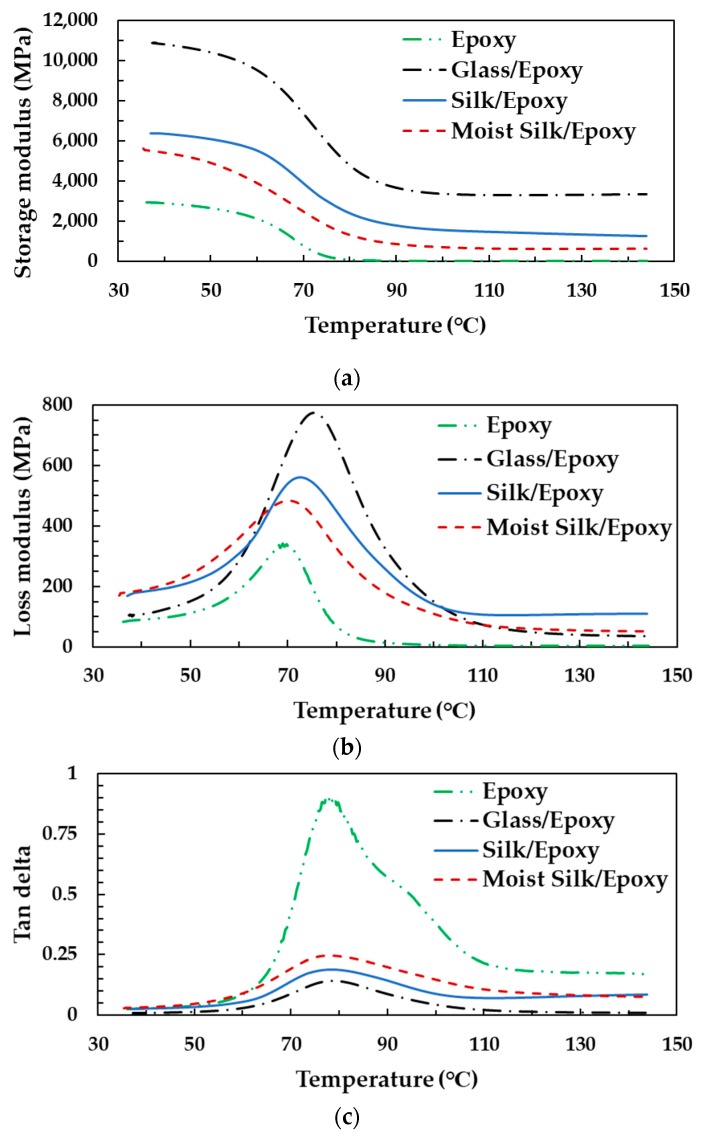
Representative changes of the (**a**) storage modulus E′, (**b**) loss modulus E″, and (**c**) tanδ for epoxy, glass/epoxy, and silk/epoxy laminates over a temperature range of 30 to 150 °C.

Figure 11 shows representative changes of the storage modulus E′, loss modulus E″, and tanδ (the ratio of loss modulus E″ to storage modulus E′) over a temperature range of 30 to 150 °C for the epoxy, glass/epoxy, and silk/epoxy laminates. Glass/epoxy showed a higher storage modulus compared to silk/epoxy and neat epoxy for all considered temperatures, which is expected since glass fibers are much stiffer than silk. Glass transition temperature was calculated using the storage modulus, loss modulus, and tanδ for all tested samples, and the obtained average values are presented in Table 3.

Silk/epoxy laminates were found to exhibit a glass transition temperature comparable, if not higher, than glass/epoxy. When measured using tanδ for instance, silk/epoxy exhibited a T_g_ of 79.5 °C compared to 78.2 °C for glass/epoxy. Comparable T_g_ values are also obtained when using both the storage and loss moduli. In addition, using silk as reinforcement was observed to increase the glass transition temperature of the epoxy resin. These findings showed that silk/epoxy laminates offer comparable glass transition temperatures to glass/epoxy, which could make silk a useful alternative to glass fibers in T_g_ sensitive applications.

### 3.10. Silk Moisture Effects on Silk/Epoxy Laminate Performance

**Figure 12:**
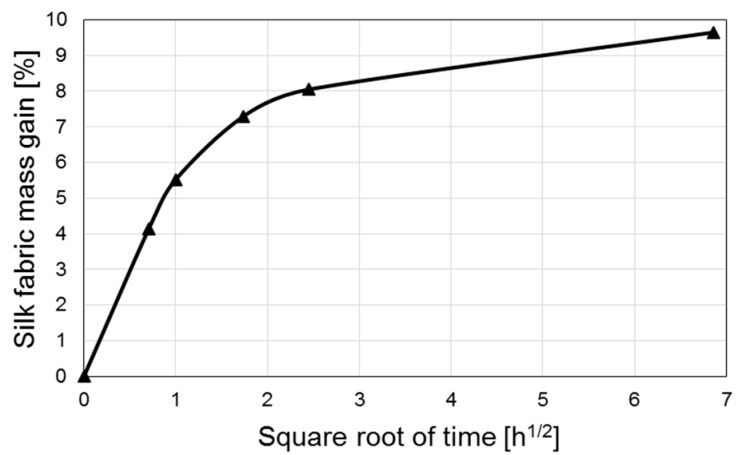
Moisture absorption curve of silk fabric exposed to a humid environment at 35 °C.

Unlike glass and other inorganic fibers that do not absorb moisture [46], organic fibers readily absorb ambient moisture, which would alter the mechanical performance of the produced composites. In order to investigate the effect of storage conditions of the silk fabrics on the mechanical performance, silk/epoxy laminates were manufactured with silk fabric containing ~10% moisture by weight. As revealed in Figure 5, this moisture content approximately corresponds to the initial weight drop observed in silk fabrics during TGA tests. Therefore, after the usual drying cycle in a vacuum oven at 50 °C, the silk fabric layers were exposed to a humid environment at 35 °C, and their weight gains were monitored. Figure 12 presents a representative moisture absorption curve of silk fabric. A moisture content of about 10% is usually reached within 48 h.

Immediately after the moisture content reached 10%, a vacuum bag lay-up was prepared and the moist fabric was infused following the same VARTM procedure used in this study. Since the fabric is exposed to vacuum within the mold for about 5 min prior to impregnation, a mock molding was performed. During this mock molding, a similar lay-up was prepared, thus exposing the moist silk fabrics to vacuum for 5 min. The fabrics were taken out of the lay-up and weighed after the 5 min vacuum period. The difference in the fabric weights was negligible, thus indicating no appreciable moisture loss during this period. The faster infusion case (S/T) was chosen as a baseline for investigating the silk moisture effects. Hence, two laminates were fabricated by infusing resin along the transverse (*x*) direction of the moist silk fabric.

Interestingly, presence of moisture in the silk fabric was observed to affect the impregnation dynamics. As presented in Table 2, the average fill time increased from 20 to 32 min due to the presence of 10% moisture in the silk fibers. Conceivable causes of this ~60% increase in fill time include accelerated cross-linking and viscosity change of the epoxy resin due to moisture. Decreased preform permeability due to slight swelling of the fabric can also slow the impregnation. Moisture-induced preform swelling combined with a slower filling also resulted in a slight increase in part thickness from 2.43 to 2.51 mm, as shown in Table 1. Moisture contact with the advancing epoxy front during impregnation and evaporation of the water during curing might also have caused the slight increase in void content from 1.1% to 1.3% (Table 2).

As presented earlier in Figure 5, the TGA behavior of laminates fabricated with dry and moist silk fabric overlap, indicating a similar thermal degradation behavior. Furthermore, dynamic mechanical analysis was performed on dry and moist silk reinforced composites. As presented in Figure 11, moisture presence in silk fabric is found to decrease the thermomechanical properties of the silk/epoxy, including storage modulus, loss modulus, and glass transition temperature. For instance, the peak loss modulus decreased from ~561 MPa for dry silk/epoxy to ~485 MPa for moist silk/epoxy. A concurrent drop from ~6500 MPa to ~5500 MPa was observed in storage modulus at 30 °C. Glass transition temperature was also lower for moist silk/epoxy regardless of the calculation method as shown in Table 3. For instance, when calculated using the loss modulus, moisture presence in the silk fabric caused the glass transition to drop from ~74 to ~72 °C. A similar drop from ~61 to ~57 °C was observed using the storage modulus.

**Figure 13:**
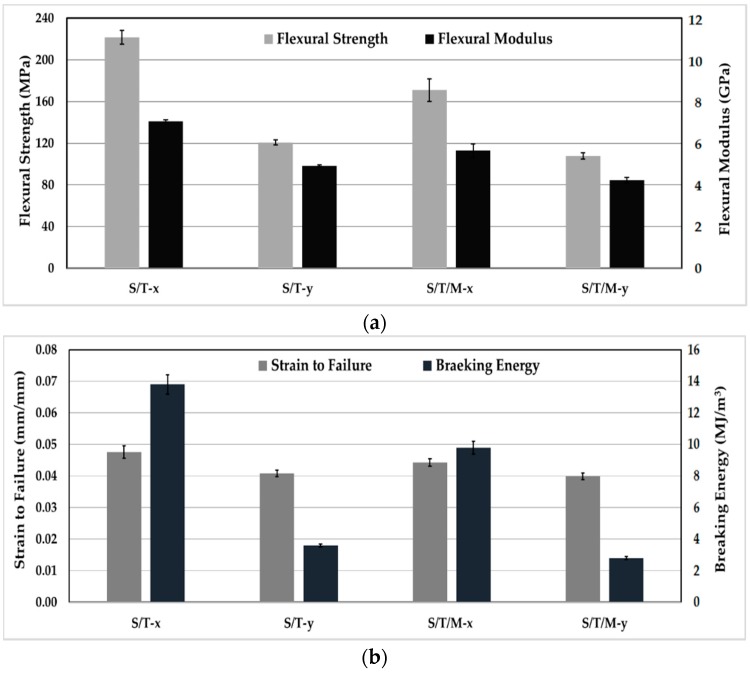

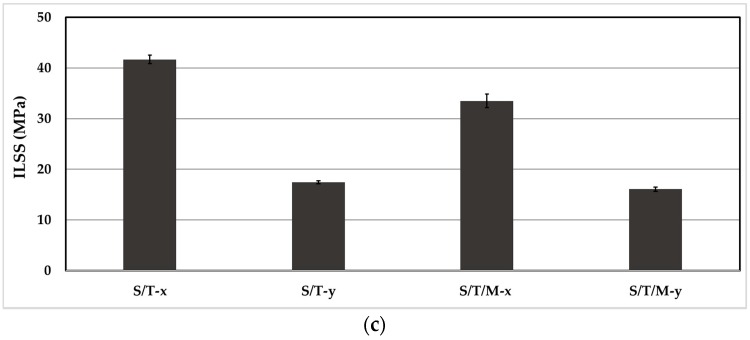
Effect of the presence of 10% moisture in the silk fabric on the mechanical performance of silk/epoxy laminates: (**a**) Flexural strength and modulus; (**b**) strain to failure and breaking energy; and (**c**) ILSS.

Additionally, silk fabric moisture was found to detrimentally affect the mechanical performance of silk/epoxy laminates, as depicted in Figure 13. The presence of 10% moisture in the silk fabric prior to fabrication was found to induce 23 and 20% reductions in specific flexural strength and modulus of the laminates along the transverse (*x*) direction, respectively. Similarly, in absolute values, moisture presence caused the flexural strength and modulus to drop respectively by 23 and 20% along the *x* direction, as depicted in Figure 13a. As depicted in Figure 13b, silk moisture also caused approximately 29 and 33% reductions in the breaking energy along both *x* and *y* directions, while the strain to failure dropped by 2 to 8%. Silk moisture also critically affected the interlaminar shear strength as shown in Figure 13c. A 20% reduction in ILSS was registered between dry and moist silk/epoxy laminates along the *x* direction. Hence, the storage conditions of the silk fabrics that are to be used in composite laminates should be carefully monitored. Drying silk fabrics to remove the absorbed moisture can significantly alleviate these adverse effects on the composite performance.

## 4. Conclusions

The processing and properties of silk/epoxy composite laminates were investigated to assess the viability of silk as a natural alternative to glass as a reinforcement in polymer composites. Vacuum-assisted resin transfer molding (VARTM) was used to manufacture composite laminates reinforced with plain weave silk.

Silk/epoxy laminates exhibited similar thermal stability, glass transition temperature, and thermomechanical properties to glass/epoxy composites. In addition, specific mechanical properties of silk/epoxy laminates were comparable to those of glass/epoxy. Silk composites even exhibited a 23% improvement over glass in specific flexural strength. In addition, applying a 300 kPa post-fill external pressure did not show a considerable effect on mechanical properties, except the breaking energy and interlaminar shear strength (ILSS). However, silk/epoxy laminates showed anisotropic mechanical properties due to the unbalanced weave pattern of the silk fabric. Therefore, developing silk fabrics with a balanced planar weave may prove beneficial when used as an ecofriendly alternative to glass reinforcement in structural composites. SEM analysis of the silk/epoxy composites revealed extensive fiber/matrix debonding. Treating the silk surface seems to be necessary for higher silk/epoxy interfacial adhesion, and for further improvement of mechanical properties.

Finally, silk fabric was found to be prone to moisture absorption from the environment, which considerably degraded the mechanical properties of the fabricated laminates. The moisture presence in silk fabric prior to laminate fabrication yielded slower fill times and caused an overall reduction in thermomechanical properties. A 10% fabric moisture content induced 23 and 20% reductions in specific flexural strength and modulus, respectively. These results stress the elevated sensitivity of silk composites to the storage conditions of the silk preforms.

## Figures and Tables

**Table 1:** Laminate designations, reinforcement types, and fabric/lay-up details. The external pressure is given in gauge pressure.

Designation	Reinforcement Type	Impregnation Direction	Fabric Condition	External Pressure (kPa)	Thickness (mm)
E	None	None	None	0	2.76 ± 0.07
G [43]	Glass Mat	Random	Dry	0	1.45 ± 0.02
S/L	Silk Woven	Longitudinal	Dry	0	2.39 ± 0.01
S/T	Silk Woven	Transverse	Dry	0	2.43 ± 0.01
S/L/P	Silk Woven	Longitudinal	Dry	300	2.22 ± 0.01
S/T/P	Silk Woven	Transverse	Dry	300	2.18 ± 0.01
S/T/M	Silk Woven	Transverse	Moist	0	2.51 ± 0.01

**Table 2:** Density, fiber content, void content, and fill time of the manufactured composite laminates (n = 10, 95% confidence interval).

Designation	Density (g/cm^3^)	Fiber Content (%)	Void Content (%)	Average Fill Time
E	1.140 ± 0.001	0	N/A	N/A
G	1.730 ± 0.010	45.7 ± 0.1	1.86 ± 0.72	3 min
S/L	1.227 ± 0.003	42.8 ± 0.1	0.66 ± 0.21	68 min
S/T	1.223 ± 0.001	43.5 ± 0.1	1.12 ± 0.06	20 min
S/L/P	1.237 ± 0.002	46.8 ± 0.2	0.53 ± 0.17	68 min
S/T/P	1.233 ± 0.002	48.4 ± 0.1	1.24 ± 0.17	20 min
S/T/M	1.226 ± 0.002	45.7 ± 0.4	1.30 ± 0.12	32 min

**Table 3:** Glass transition temperature, T_g_, of silk/epoxy compared to glass/epoxy and neat epoxy.

Designation	T_g_ by Storage Modulus (°C)	T_g_ by Loss Modulus (°C)	T_g_ by tanδ (°C)
E	59.9	69.6	78.4
G	59.9	75.2	78.2
S/T	60.9	74.0	79.5
S/T/M	57.4	71.9	79.3
